# Evaluation of alkali residue-stabilized soil in road construction with optimization of mechanical properties and environmental risk assessment

**DOI:** 10.1371/journal.pone.0341304

**Published:** 2026-03-13

**Authors:** Jing Hua, Wenyi Xie, Guangxia Liu, Qun Li, Yan Zhou, Ruijie Che, Fenghe Wang, Xiaowei Xu, Chang Liu

**Affiliations:** 1 Nanjing Institute of Environmental Science, Ministry of Ecology and Environment of China, Nanjing, China; 2 Nanjing University of Science and Technology, Nanjing, China; University of Sindh, PAKISTAN

## Abstract

The effective utilization of alkali residue (AR) is a central issue and an urgent challenge for the green transformation of the soda ash industry. While research has primarily focused on the mechanical performance of AR-based materials, their long-term environmental risks, particularly under dynamic conditions, have been relatively overlooked. This study evaluates AR-stabilized soil for road construction by simultaneously optimizing its mechanical properties and conducting a comprehensive environmental risk assessment for shallow groundwater contamination. Through laboratory tests, we identified the AR substitution rate that maximizes the California Bearing Ratio (CBR) and water stability. To address the limitations of traditional static leaching tests, we integrated material degradation and contaminant transport models to dynamically simulate pollutant release and migration under realistic, non-steady conditions. Our findings reveal that an AR substitution rate between 10% and 50% yields optimal mechanical performance. However, environmental risk analysis indicates a significant increase in risk beyond 35%, with manganese (Mn) and nickel (Ni) exposure concentrations potentially exceeding Class III groundwater quality standards. Sensitivity analysis confirms the AR substitution rate as the most critical factor influencing environmental risk. Therefore, we recommend strictly controlling the AR substitution rate to ≤35% in road construction, providing a science-based, dual-criteria (mechanical and environmental) guideline for the safe and sustainable utilization of industrial waste in geotechnical engineering.

## Introduction

Soda ash is an indispensable basic chemical raw material in the national economy, widely used in industries such as medicine, papermaking, metallurgy, glass, and textiles [1]. By 2023, the production capacity of soda ash in China had exceeded 35 million tons, with domestic production capabilities stabilizing [2]. The predominant methods for producing soda ash encompass the ammonia-soda process, the combined soda process, and the natural soda process [3]. Notably, the ammonia-soda process constitutes approximately 45% of the total production capacity [2]. However, this method generates a significant amount of AR during production. Characterized by fine particles, high moisture content, and the presence of potentially harmful substances such as heavy metals Mn, Ni, and Ba, the recycling and utilization of these residues present considerable challenges, with utilization rates remaining below 5% [4]. As a result, a substantial amount of AR is stockpiled locally, leading to the wastage of land resources and posing risks of environmental pollution and safety hazards, including landslides and slope failures, which have resulted in serious social issues [2,5]. In line with the Guiding Opinions on the Comprehensive Utilization of Bulk Solid Wastes During the 14th Five-Year Plan Period, there is a plan to increase the comprehensive utilization rate of bulk solid waste to 60%, with the aim of reducing existing waste stockpiles and achieving harmless treatment. In 2022, the Ministry of Industry and Information Technology and the National Development and Reform Commission jointly issued guidelines that emphasize the acceleration of green and low-carbon development and promote the environmental governance of waste residues and wastewater generated during the ammonia-soda production process. Against this backdrop, the effective resource utilization of AR has emerged as a crucial topic and an urgent task for the green transformation and high-quality development of the soda ash industry.

The resource transformation of AR has emerged as a research hotspot in recent years. AR primarily consists of elements such as calcium, magnesium, silicon, and aluminum, rendering it suitable for producing non-fired bricks, concrete, and other building materials, as well as for use as backfill material in mines and roads. Lin Yonghui et al. [6] found that AR can serve as an alkaline activator for slag powder, achieving optimal physical properties, mechanical strength, and microstructure of activated slag cement-based materials when the AR admixture ratio is 16%, with a compressive strength of 34.1 MPa and a flexural strength of 6.9 MPa at 28 days. Zhao Xiaoqing et al. [7] observed in their study that increasing the AR admixture ratio to 70% in stabilized soil significantly enhances the material’s mechanical properties, achieving a CBR of 78.5% and a resilient modulus of 600 MPa, thereby demonstrating excellent stability and mechanical performance. Liu Jingjing et al. [8] determined that AR used as a stabilizer for zinc-contaminated soils effectively reduces the mobility and toxicity of heavy metals. After stabilization with 20% AR, the leaching rate of Zn decreased by 85%. However, freeze-thaw cycles significantly degrade the stabilization effect. After 10 freeze-thaw cycles, the leaching concentration of Zn increased markedly, with the leaching rate rebounding to 40%. This indicates the complexity of pollution release from solid waste-based materials in environmental settings.

Although research on alkali residue utilization has made progress, a critical gap persists in the holistic evaluation of AR-stabilized soil for engineering applications. Prior studies have largely operated in silos: some focus exclusively on enhancing mechanical properties, while others rely on simplified static leaching tests for environmental assessment. Notably, an integrated approach that concurrently optimizes mechanical performance and evaluates dynamic environmental risks—employing probabilistic methods similar to those advanced in geotechnical risk analysis—has not been established for AR-stabilized soil [9–12]. This lack of integration means that potential peak environmental risks under non-steady conditions, such as those driven by rainfall infiltration and groundwater flow, may be overlooked by conventional static assessments. Consequently, the long-term environmental safety of AR reuse in large-scale projects like road construction remains uncertain. Therefore, developing a comprehensive framework that bridges mechanical and dynamic environmental evaluations is essential for the safe and sustainable application of this industrial by-product.

To bridge this critical gap and advance the sustainable use of industrial wastes in civil engineering, this study proposes an integrated assessment framework for alkali residue stabilized soil in road construction, aiming to balance engineering performance with environmental safety. The research first determines the optimal AR substitution rate by evaluating the mechanical properties, specifically the California Bearing Ratio and water stability coefficient, of stabilized soil through standardized laboratory tests. Subsequently, to overcome the limitations of static leaching evaluations, a dynamic and probabilistic environmental risk assessment is conducted for shallow groundwater contamination by coupling contaminant release models with transport models. Finally, sensitivity and uncertainty analyses are performed to identify the dominant factors influencing environmental risk, leading to science-based recommendations for the safe application of AR-stabilized soil in road infrastructure.

## Materials and methods

### Sample preparation and testing analysis

Lianyungang City in Jiangsu Province is one of China primary soda ash production bases, where traditional soda ash production primarily uses the ammonia-soda process. During this process, approximately 0.3 to 0.6 tons of AR are generated per ton of soda ash produced. Over several decades, this has resulted in a local AR stockpile exceeding 20 million m^3^. This study focuses on desalinated AR from this stockpile. No permits were required for this work, as the material was obtained from a non-sensitive industrial site and all experiments were conducted under controlled laboratory conditions without any field disturbance.

CBR Test: The CBR test was conducted according to the bearing ratio test method specified in the Test Methods of Soils for Highway Engineering (JTG 3430−2020) [13]. The test specimens were prepared at the optimal moisture content and compacted using the Heavy Type II-2 compaction method, with six replicate samples prepared for each mixture. Additionally, the soaking time for the specimens was set at 96 hours.

Dry-Wet Cycle Test [13]: Cylindrical specimens (Φ50 mm × 50 mm) were prepared by mixing alkali residue (AR) and clay at predetermined mixtures. After 7 days of curing, six replicate specimens were prepared for each mixture in both the dry-wet cycle group and the control group. The dry-wet cycle group underwent five 48-hour cycles (24 h soaking + 24 h drying), while the control group was maintained under stable conditions. Subsequently, both groups were soaked for 24 hours prior to testing. The unconfined compressive strength was measured for all specimens, and the water stability coefficient was calculated as the ratio of the average unconfined compressive strength of the dry-wet cycle group to that of the control group.

Contaminant Detection Analysis: After breaking down the AR-stabilized soil samples, contaminant content and leachability were quantitatively analyzed following the pretreatment and testing methods specified in GB 5085.3 [14] and GB 5085.6 [15]. For each AR substitution ratio, three replicate samples were tested, and the mean values were used as input for the environmental risk model.

### Characterization of contaminant risk

#### Characterization of pollutant release.

During the utilization of AR in road construction, pollutants are leached and released into the underlying soil and groundwater environment by rainfall infiltration. As rainfall continues to infiltrate, the pollutant components in the solid waste are continuously leached and released. This study employs an exponential decay source model to describe the change in pollutant release concentration within the source strength, represented by Equations (1) and (2) [16] as follows:


$${\text{C}}_{\text{t}}={\text{C}}_{\text{0}}\times {\text{e}}^{-\lambda \text{t}}$$ (1)

$$\lambda =\frac{\alpha {\text{C}}_{\text{0}}}{df\rho {\text{C}}_{\text{sw}}}$$ (2)

where $C_{t}$ represents the release concentration of pollutants in the source strength at any time *t* in mg/L; $C_{\text{0}}$ is the initial concentration of pollutants in the leachate in mg/L; α is the groundwater infiltration rate in m/a; *d* is the thickness of the roadbase layer in m; ρ is the density of the roadbase layer in kg/L; *f* is the volume fraction of AR in the roadbase layer and is dimensionless; ρ is the density of the roadbase layer in kg/L; and $C_{sw}$ is the maximum effective release amount of pollutants in mg/kg.

#### Characterization of contaminant migration and dispersion.

The natural attenuation process can primarily be divided into two steps. The first step is the vertical leaching process (LF) where heavy metals in AR-stabilized soil are leached out by rainfall into groundwater. The second step involves the lateral advection and dispersion process (DAF), where contaminants enter the groundwater and then migrate, dilute, and disperse as the groundwater flows towards observation wells. Based on the exposure pathways of characteristic contaminants in stabilized soil, the concentration of contaminants in groundwater after dilution and attenuation can be represented by Equation (3) [17]:


$${\text{c}}_{\text{gw}}={\text{c}}_{\text{w}}\times \text{LF}\times \text{DAF}$$ (3)

where $c_{gw}$ represents the concentration of pollutants in groundwater at the observation well in mg/L; $c_{w}$ represents the content of pollutants released from the pollution source in mg/kg; LF is the soil leaching and dilution factor in kg/L; and DAF is the groundwater dilution and attenuation coefficient, which is dimensionless.

To calculate LF during the leaching process where contaminants from stabilized soil migrate and disperse into groundwater, a model based on the leaching factor for contaminant migration into groundwater as specified in Technical Guidelines for Risk Assessment of Soil Contamination of Land for Construction (HJ 25.3–2019) [17] is used. This calculation takes into account various influencing factors such as soil density, permeability, contaminant partition coefficient, thickness of the mixing zone, and moisture content in the unsaturated zone. The dilution factor LF for the leaching process can be represented by Equations (4) to (6) [17]:


$$LF=\frac{{LF}_{spw-gw}}{K_{sw}}$$ (4)

$${LF}_{spw-gw}=\frac{\text{1}}{\text{1}+\frac{U_{gw}\times \delta_{gw}}{I\times W}}$$ (5)

$$K_{sw}=\frac{\theta_{ws}+K_{d}\times \rho_{b}+H\times \theta_{as}}{\rho_{b}}$$ (6)

where *LF* represents the leaching factor for pollutants migrating from roadbase materials into groundwater in kg/L; ${LF}_{spw-gw}$ is the leaching factor for pollutants migrating from soil pore water into groundwater, which is dimensionless; $K_{sw}$ is the partition coefficient of pollutants between soil and water in m^3^/kg; $U_{gw}$ is the Darcy velocity of groundwater in m/s; $\delta_{gw}$ is the thickness of the groundwater mixing zone in m; *I* is the infiltration rate of water in the soil in m/s; *W* is the width of the road in m; $K_{d}$ is the partition coefficient of pollutants between soil solid phase and water in m^3^/kg; $\theta_{ws}$ is the volumetric ratio of pore water in unsaturated soil, which is dimensionless; $\theta_{as}$ is the volumetric ratio of pore air in unsaturated soil, which is dimensionless; $\rho_{b}$ is the bulk density of soil in kg/L; and *H* is the Henry’s constant for the pollutant, which is dimensionless.

#### Dilution and attenuation factor (DAF).

The dilution and attenuation of pollutants in groundwater are controlled by processes such as the dilution effect of groundwater flow (i.e., convection) and the attenuation effect of the subsurface medium (i.e., degradation, dispersion, and retardation). In homogeneous and isotropic soil-water systems, the transport process as well as the dilution and attenuation effects can be modeled using the one-dimensional advective-dispersive equation, as shown in Equation (7) [18,19]:


$$\frac{\partial C}{\partial t}=D_{L}\frac{\partial^{\text{2}}c}{\partial x^{\text{2}}}-\frac{v}{n}\frac{\partial c}{\partial x}-R\gamma C$$ (7)

where *C* represents the concentration of pollutants at a distance *x* and time *t*, measured in mg/L; *x* denotes the distance along the direction of groundwater flow, measured in m; *u* is the groundwater velocity, measured in m per second (m/s); *n* represents the effective porosity, which is a dimensionless quantity; *R* is the retardation factor; *γ* is the first-order decay rate, measured in seconds (s); and $D_{L}$ is the hydrodynamic longitudinal dispersion coefficient, measured in m per second (m/s).

The analytical solution for a continuously injecting point source is given by Equation (8):


$$DAF=\frac{\text{1}}{\text{2}}erfc\left(\frac{x-ut}{\text{2}\sqrt{D_{L}t}}\right)+\frac{\text{1}}{\text{2}}{\text{e}}^{\frac{ux}{D_{L}}}erfc\left(\frac{x+ut}{\text{2}\sqrt{D_{L}t}}\right)$$ (8)

where *DAF* represents the groundwater dilution and attenuation factor, which is a dimensionless quantity; *x* denotes the distance along the direction of groundwater flow, measured in m; *u* is the groundwater velocity, measured in m per day (m/d); $D_{L}$ is the longitudinal dispersion coefficient, measured in square meters per day (m^2^/d); and *erfc()* is the complementary error function.

### Characterization of uncertainty

The Monte Carlo method was employed to characterize the impact of parameter uncertainties on the results, with 10,000 iterations performed to ensure result stability and accuracy. Meteorological parameters, such as rainfall, were primarily based on data from the 2022 China Statistical Yearbook. Groundwater-related parameters, including the water content ratio in the unsaturated zone, soil bulk density, thickness of the groundwater mixing zone, Darcy velocity of groundwater, porosity, and soil permeability, were adopted using the recommended values from HJ 25.3–2019. Road-related parameters were as follows: the thickness of the subgrade layer was selected according to domestic traffic highway standards, ranging from 0.3 to 1.2 m; the width W of the road ranged from 3.5 to 30 m; and the horizontal distance from the road (exposure source) to the observation well was set at 100 meters. The regulation that the distance from the centerline of the highway to the water source should not be less than 100 meters, as specified in the Design Specifications of Highway Environmental Protection (JTG B04-2010) [20], was used as the reference basis. All simulations and statistical analyses were performed using MATLAB R2023a.

## Results and discussion

### Mechanical properties of AR-stabilized soil

**Fig 1:**
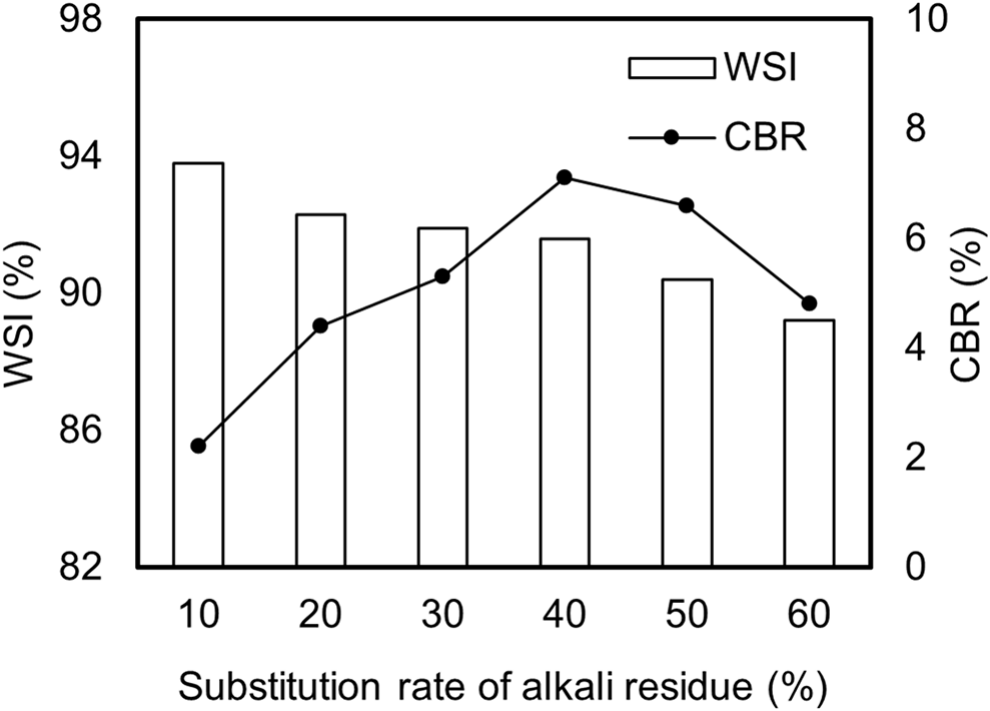
Variation of CBR value and WSI with AR substitution rate.

In this study, systematic experiments were conducted to explore the impact of AR substitution rates on the CBR and water stability index (WSI) of stabilized soil materials. Fig 1 shows that the CBR value initially increases with AR substitution, peaking at 7.1 when the substitution rate reaches 40%, before declining at higher rates. This suggests that moderate AR incorporation improves load-bearing capacity by optimizing particle gradation and enhancing compaction density. In contrast, excessive AR disrupts cohesion, leading to structural weakening. Research shows that while cohesive soil has good cohesion, it lacks large particles for support and tends to form loose structures [21]. Conversely, AR can build a more stable framework but has insufficient cohesion when used alone, making it difficult to form high-strength materials [22]. Therefore, mixing AR with cohesive soil to form stabilized soil materials can optimize particle gradation, allowing fine particles to fill the gaps between AR sand particles, thereby enhancing material density and strength. During compaction, the interaction between particles strengthens, aiding in better gap filling and improving compaction effectiveness, which in turn reinforces the overall strength of the material.

Concurrently, the WSI gradually decreases from 93.8% to 89.2% as the AR substitution rate rises to 60%, falling below the 90% threshold required by JTG D30-2015 [23]. This decline reflects the detrimental effect of repeated dry-wet cycles on internal integrity, as cyclic expansion and contraction induce microcracking and particle loss, particularly in high-AR mixtures. In conclusion, to ensure a balance between the mechanical properties and water stability of stabilized soil materials, it is recommended to control the AR substitution rate between 10% and 50%. This conclusion is supported by previous studies, such as the research by Ma Jiaxiao et al. [24], which showed that when the AR substitution rate approaches 50%, the mechanical properties of AR-soil significantly improve, with unconfined compressive strength, cohesion, and internal friction angle reaching 0.43 kPa, 74 kPa, and 32°, respectively, demonstrating excellent engineering applicability. Bai Xiaoyu et al. [25] found that as the AR substitution rate increases, the strength of AR-soil first increases and then decreases, attributed to the reaction between calcium oxide in fly ash and aluminum oxide in AR forming hydration products that provide binding effects; however, an excessively high AR ratio leads to increased porosity and weakened binding effects, thus reducing overall strength.

### Environmental risk and evolution of AR-stabilized soil

**Fig 2:**
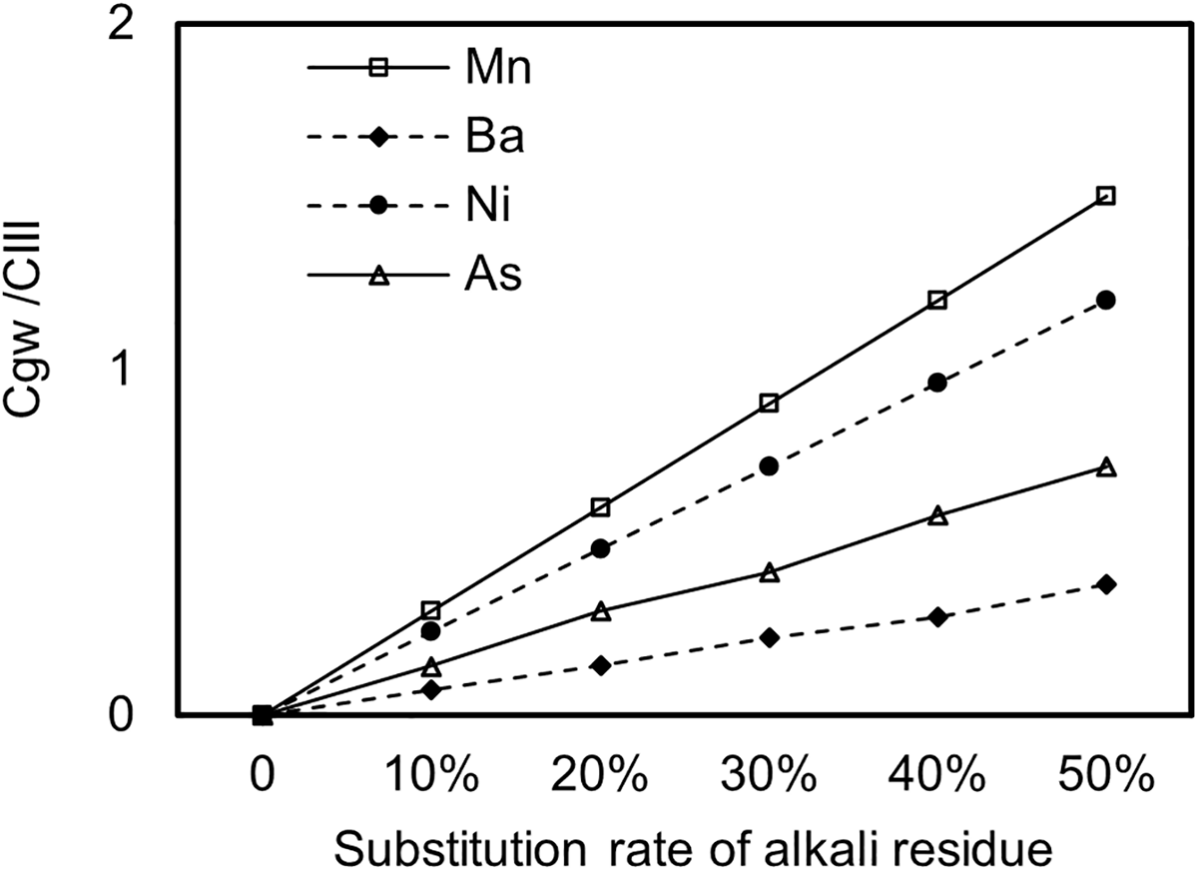
Changes in pollutant standard occupation rates of Ba, Ni, As, and Mn with AR substitution rates.

Leaching experiment results show that no organic compounds were detected in the leachate from AR, while the detection rates of heavy metals such as Hg and Cd were both below 5%, with extremely low leaching concentrations. However, heavy metals like Ba, Ni, As, and Mn were detected in all samples, with relatively higher leaching concentrations. Therefore, these four heavy metals were identified as key contaminants for subsequent analysis and assessment. Fig 2 illustrates the changes in the ratio of exposure concentrations of Ba, Ni, As, and Mn to the Class III water quality standard limits specified in GB/T 14848 under different AR substitution rates. When the pollutant standard occupation rate is less than 1, it indicates that the concentration of harmful components in groundwater from observation wells meets the requirements for drinking water standards. According to Fig 2, the standard occupation rate for Mn drops to 1 when the AR substitution rate is approximately 35%, indicating that the maximum AR substitution rate for Mn is 35%. Similarly, the maximum AR substitution rate for Ni is 42%; whereas for Ba and As, even at an AR substitution rate of 50%, the standard occupation rate remains below 1, indicating lower environmental risks for Ba and As in AR-stabilized soil. The higher risk of Mn and Ni is attributed to their combination of relatively high initial leaching concentrations and stringent groundwater quality limits.

**Fig 3:**
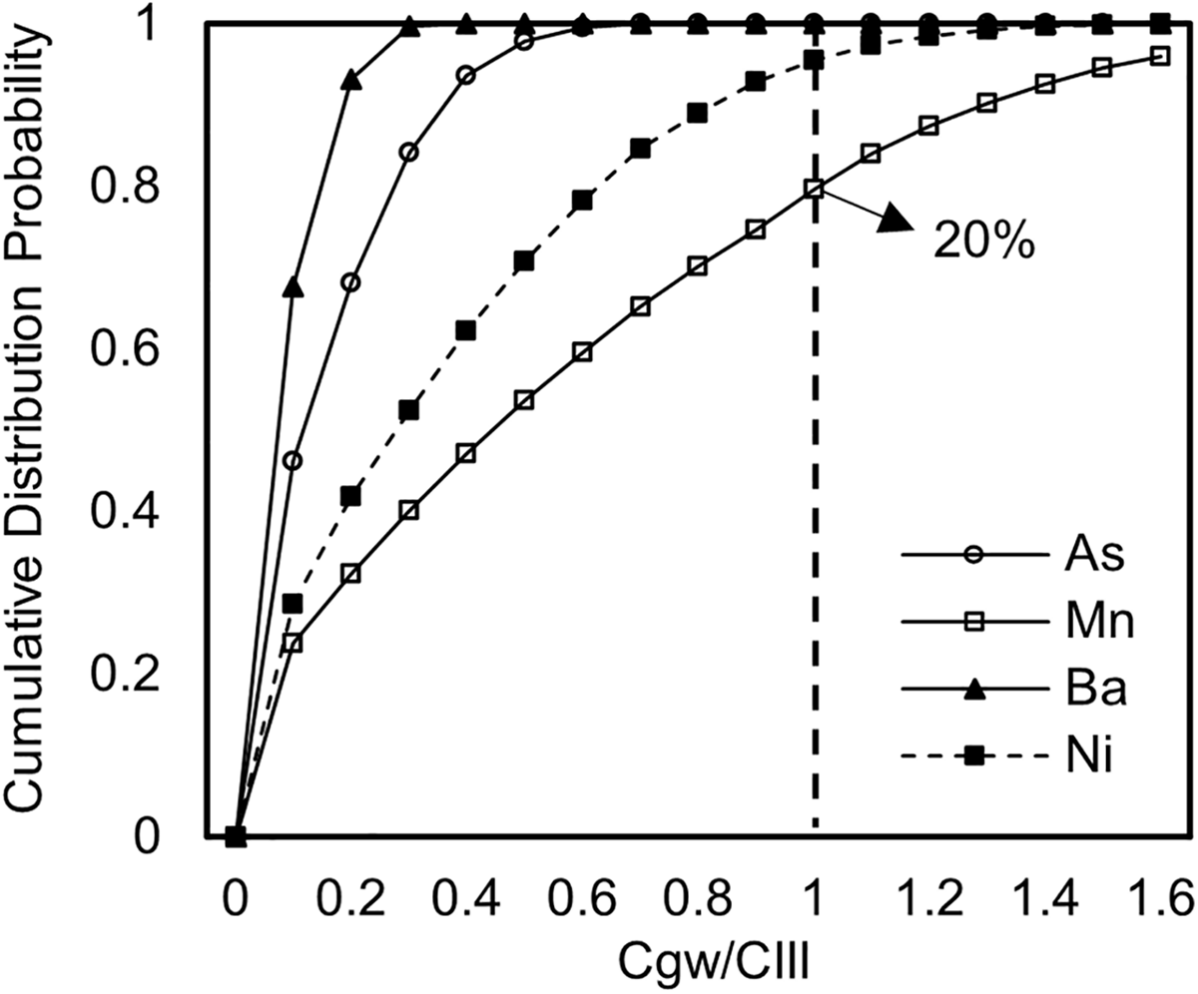
Cumulative frequency distributions of contaminant concentrations under uncertainty conditions.

Using the Monte Carlo method, the cumulative frequency distributions of contaminant concentrations under uncertainty conditions are shown in Fig 3. By comparing these distributions with the Class III groundwater quality limits, the exceedance probability and exceedance multiples were calculated. From Fig 3, it can be observed that the exposure concentrations of As are all below its corresponding Class III groundwater limit of 0.01 mg/L, with a standard occupation rate ranging from 0 to 0.55, all below 1, indicating that the risk is within a controllable range. The exposure concentrations of Ba are all below its corresponding Class III groundwater limit of 0.7 mg/L, with a standard occupation rate ranging from 0 to 0.29, all below 1, indicating that the risk is within a controllable range. The ratios of Mn and Ni concentrations after groundwater migration and transformation to the Class III groundwater quality limits have the potential to exceed 1. Specifically, the probability of Mn exceeding the Class III groundwater quality limit is 20%, with the exposure concentration of Mn being 1.39 times the Class III groundwater quality limit. The probability of Ni exceeding the Class III groundwater quality limit is 4.2%, with the exposure concentration of Ni being 1.2 times the Class III groundwater quality limit. These findings highlight that while Ba and As pose minimal risk, Mn and Ni require strict control of AR substitution rates, ideally not exceeding 35 percent, to mitigate probabilistic exceedances of groundwater quality standards.

**Fig 4:**
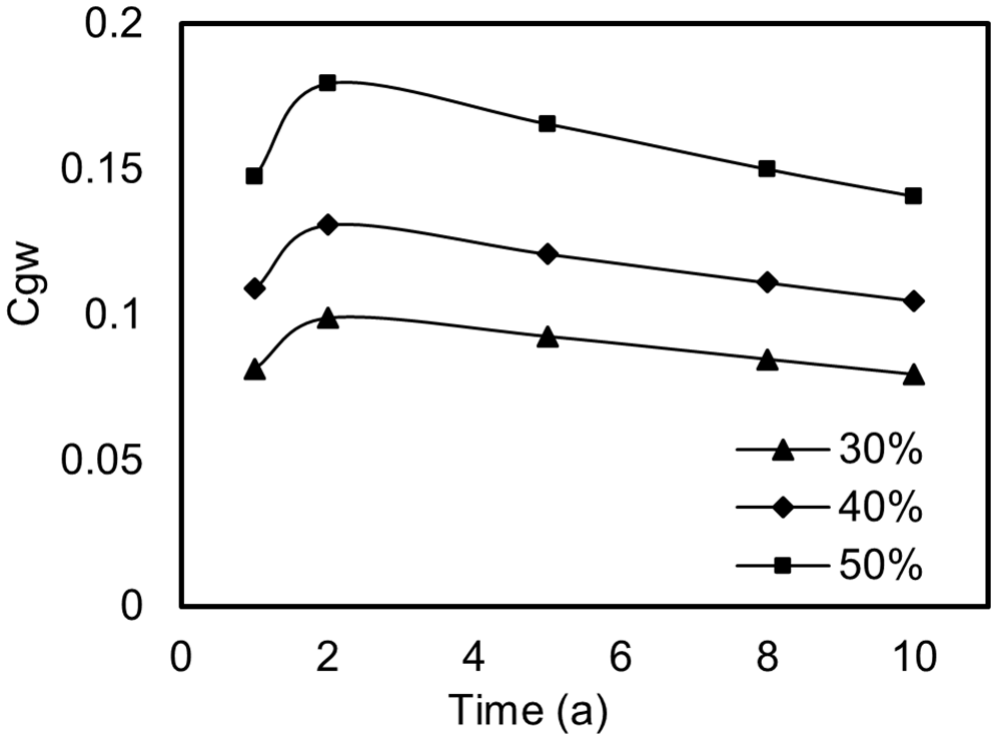
Trends in Mn concentration in groundwater over time for AR substitution rates of 30%, 40%, and 50%.

In the realm of road engineering, the integration of source strength index decay models with contaminant migration models provides a profound insight into the dynamic shifts in shallow groundwater contamination attributed to Mn emanating from AR-stabilized soil. Fig 4 delineates the temporal trends in groundwater Mn concentrations corresponding to AR substitution rates of 30%, 40%, and 50% within stabilized soil matrices. Initially, Mn concentrations surge to a peak before gradually subsiding. For instance, at a 40% substitution rate, the application of AR-stabilized soil as a subgrade bedding material leads to a rapid infiltration of Mn into the groundwater under the impetus of rainfall. Within the first year of deployment, the Mn concentration at the exposure point in the groundwater ascends to 0.108 mg/L; by the second year, this fig escalates to 0.13 mg/L, surpassing China’s Class III groundwater quality standard limit by 1.3 times. Subsequently, owing to the attenuation of source strength and the natural processes of dilution and degradation, the Mn concentration at the exposure point in the groundwater commences a decline.

It is imperative to note that despite the burgeoning application of recycled solid waste products, such as AR-stabilized soil, in road construction, the extant research on their protracted environmental repercussions remains scant. Conventional environmental risk assessments predominantly hinge on static leaching experiments, which involve a direct comparison of contaminant leachate concentrations with established standard limits. This approach may inadvertently disregard the intricate release dynamics and peak risks inherent in real-world environmental contexts. For example, Luo Zhongtao et al. [26] examined the characteristics of high-temperature melting and the formation of reconstructed slag through the admixture of blast furnace slag with municipal solid waste incineration fly ash. However, their assessment of environmental risk was confined to comparing the leaching concentrations of zinc, lead, copper, and chromium with standard limits, without adequately accounting for the long-term environmental behavior and potential accumulation effects of these heavy metals. Similarly, Li Bing et al. [27] conducted an exhaustive evaluation of the environmental risks associated with red mud-modified phosphogypsum-based self-leveling mortar, advocating for its viability as an innovative building material. Nonetheless, their study fell short in considering the migration and transformation pathways of pollutants like chromium and cadmium within the environment. Furthermore, Bai Yuejiao et al. [28] explored permeable concrete paving bricks fabricated through the co-processing of electrolytic manganese residue, red mud, and fly ash. Their findings indicated that by fine-tuning the production process and mix proportions, the leaching toxicity of heavy metals such as Ni, Cu, Zn, and Mn could be brought in line with regulatory standards. Bai Yuejiao et al. [28] also underscored the significance of incorporating dynamic models capable of simulating the long-term release and transformation of heavy metals in solid waste materials, thereby spotlighting the imperative for more precise risk assessments.

To achieve a more accurate appraisal of the environmental impact of waste-derived building materials, it is incumbent to embrace a more holistic approach that evaluates their environmental footprint across the entire lifecycle. This encompasses the establishment of more refined mathematical models designed to simulate the release and migration diffusion of contaminants, while factoring in a spectrum of potential chemical reactions and physical processes. Additionally, it entails the augmentation of monitoring and long-term tracking initiatives to ensure the timely detection and mitigation of potential environmental pollution issues.

### Sensitivity and uncertainty analysis

Environmental risk assessment models face the primary challenge of non-uniformity and complexity in input parameters, leading to exposure concentrations calculated using model preset values that may significantly differ from actual conditions [29]. Comprehensive field measurements can enhance accuracy but are costly, inefficient, and some parameters are difficult to obtain [30]. Consequently, analyzing the sensitivity and uncertainty of model parameters is essential to identify key parameters that significantly influence simulation outcomes, thereby prioritizing them in field testing.

**Fig 5:**
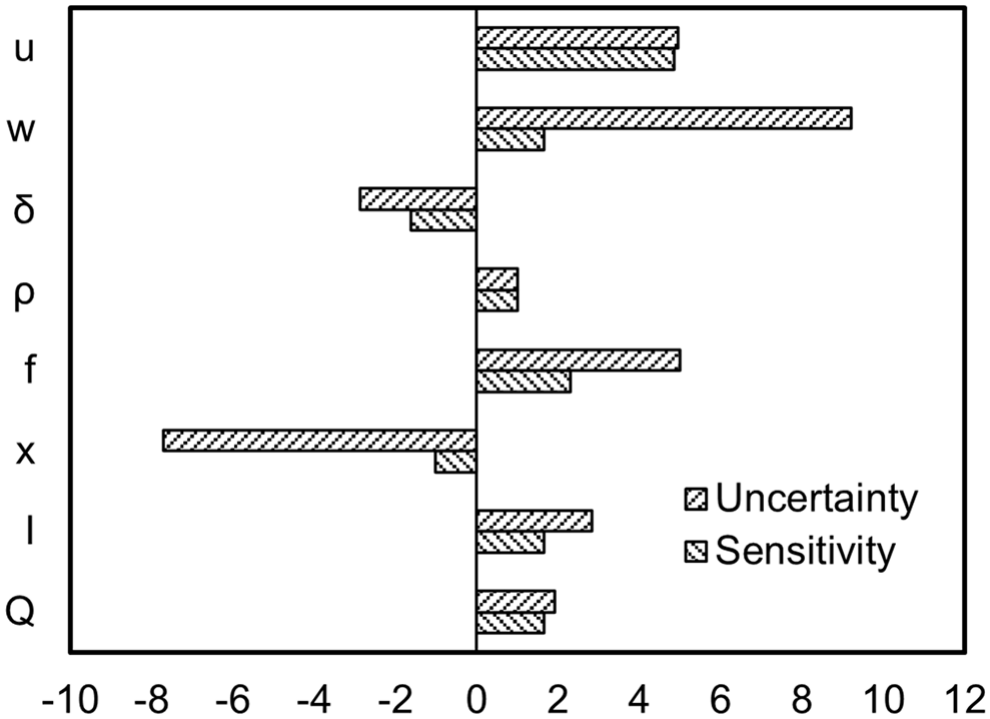
Uncertainty and sensitivity analysis.

Sensitivity analysis evaluates the impact of parameters on model outputs by adjusting them to ±25% of their default values. Uncertainty analysis quantifies the uncertainty of model predictions by establishing reasonable ranges for parameters. Together, these analyses ensure the validity and reliability of the model. Fig 5 presents the sensitivity and uncertainty analysis results for parameters involved in the process of pollutants leaching from AR-stabilized soil in road applications, entering groundwater, and migrating downstream. As depicted in Fig 5, during the pollutant release phase, the AR substitution rate and annual rainfall exhibit relatively high sensitivities, at 2.3 and 1.65 times, respectively, while the density of stabilized soil shows minimal sensitivity, at approximately 1.0 times. The density of stabilized soil primarily affects its mechanical properties and has a negligible impact on pollutant release. When considering parameter uncertainty, the AR substitution rate markedly influences exposure concentrations, with the upper limit substitution rate resulting in an exposure concentration nearly five times that of the lower limit. During the leaching and diffusion phases, the sensitivity of groundwater flow velocity, road width, and mixing zone thickness is relatively high, at 4.85, 1.66, and 1.62 times, respectively. When considering parameter uncertainty, the impact of road width and buffer distance is particularly significant, with uncertainties of 9.2 and 7.7 times, respectively. This suggests that when utilizing AR-stabilized soil in road construction, it should be avoided in areas where residents draw groundwater, and it is preferable to use it in the subbase of narrower roads. Future field monitoring campaigns will be essential to validate the model predictions and refine parameter uncertainties under real-world hydrogeological and climatic conditions.

Sensitivity analysis reveals that the AR substitution rate, rainfall, groundwater flow velocity, and road width are key factors influencing the environmental risk assessment of AR-stabilized soil. When conducting risk assessments and devising pollution control strategies and intervention measures for AR-stabilized soil, it is imperative to consider the impact of these parameters comprehensively, while also focusing on the potential environmental threats posed by the total amount of heavy metals in AR, their different chemical forms, and bioavailability. This approach enables more precise assessment and refined management. The application of Monte Carlo simulation methods, by accounting for the variability and uncertainty of input parameters, provides a more comprehensive and probabilistic approach to pollution risk assessment for AR-stabilized soil. Compared to traditional deterministic pollution risk assessments, the probabilistic pollution risk assessment coupled with Monte Carlo simulation effectively mitigates uncertainties due to limited sample data, providing quantifiable indicators for the uncertainty and variability of AR-stabilized soil pollution. This method facilitates a comprehensive understanding of the overall environmental pollution risks in a region, thereby offering more detailed and comprehensive information for scientific research and risk management.

## Conclusions

This study developed and applied an integrated evaluation framework for alkali residue (AR)-stabilized soil in road construction, with the core contribution being the establishment of a combined mechanical-environmental evaluation framework that quantitatively links engineering performance with dynamic environmental risk, thereby overcoming the limitations of isolated assessments and providing a science-based, dual-criteria approach for the sustainable reuse of industrial by-products; a critical finding is the inherent trade-off between mechanical strength and environmental safety, where higher AR substitution rates (35%–40%) enhance early strength but significantly increase the peak concentration and risk probability of contaminant leaching, implying that for projects requiring higher strength, such as heavy traffic roads, the use of such substitution rates should be accompanied by enhanced environmental mitigation measures—including the installation of impermeable liners, increased buffer distances from sensitive receptors, or the incorporation of reactive barriers—to manage the elevated leaching risk, thus shifting the practical recommendation from a rigid cut-off to a flexible, risk-informed management strategy; future research should focus on field-scale validation of this framework, integration of advanced geochemical modeling to refine contaminant fate predictions, and comprehensive lifecycle assessment (LCA) to evaluate the net environmental benefit, which will further strengthen the framework’s robustness and support its broader adoption in sustainable geotechnical engineering and policymaking.
